# Anodic Fabrication of Ti-Ni-Si-O Nanostructures on Ti10Ni5Si Alloy

**DOI:** 10.3390/ma12081315

**Published:** 2019-04-23

**Authors:** Ting Li, Dongyan Ding, Nan Li

**Affiliations:** 1 Handan Institute of Innovation, Peking University, Handan 056000, China; litingstar@sjtu.edu.cn (T.L.); lin@pkuhd.cn (N.L.); 2 Institute of Electronic Materials and Technology, School of Materials Science and Engineering, Shanghai Jiao Tong University, Shanghai 200240, China

**Keywords:** titanium alloy, anodization, nanostructures

## Abstract

Ti-Ni-Si-O nanostructures were synthesized on Ti10Ni5Si alloy through an electrochemical anodization in electrolyte solutions containing ammonium fluoride (NH_4_F). The anodic oxide structures were affected by the electrochemical anodization parameters, including the electrolyte viscosity, water content, anodization potential and anodization time. Using an anodization potential of 40 V for 90 min in an ethylene glycol/glycerol electrolyte with 3 vol.% deionized water, highly ordered self-organized nanotube arrays were obtained in the α-Ti phase region of the alloy substrate, with an average inner diameter of 70 nm and a wall thickness of about 12 nm. Self-organized nanopore structures with an average pore diameter of 25 nm grew in the Ti_5_Si_3_ phase region. Only etching pits were found in the Ti_2_Ni phase region. The Ti-Ni-Si-O nanostructures were characterized using scanning electron microscopy and energy dispersive spectroscopy. In addition, a formation mechanism of different nanostructures was presented.

## 1. Introduction

Titanium and titanium alloys have been widely used due to their excellent properties, such as their good chemical stability, biocompatibility, good corrosion resistance and high thermal stability [1]. Furthermore, titanium could be used to form a TiO_2_ layer, which has been widely used in solar cells [2,3], semiconductors [4], photocatalysis [5,6] and gas sensors [7]. TiO_2_ nanotubes can be fabricated through many routes, like anodization [8], sol-gel [9], template [10] and electrophoretic deposition (EPD) [11]. Among these routes, the anodization of titanium in F^—^-containing electrolytes is the most simple and low-cost way to form highly ordered self-organized TiO_2_ nanotube arrays. This route makes it possible to precisely control the surface morphologies and structures of TiO_2_ as desired [12].

During the electrochemical anodization of titanium, several fabrication parameters, including the electrolyte composition, anodization potential, anodization time, temperature, viscosity, water content and pH value of the electrolytes, greatly affect the surface features of the anodic oxide nanostructure. It was reported that in a NH_4_F-based glycerol electrolyte containing a small amount of H_2_O, self-organized TiO_2_ nanopore arrays on Ti foil were successfully obtained [13]. The surface morphologies, dimensions and pore density were strongly influenced by the applied potential and anodization temperature. Highly ordered TiO_2_ nanopore arrays were obtained at the applied potentials between 30 V and 70 V, and at an anodization temperature below 20 °C. When the anodization temperature was above 30 °C, however, the nanopore structure became irregular at 50 V, and remarkable damage was even observed at 70 V. Cai et al. [14] reported that by adjusting the solution’s pH value to 4.5, well-arranged TiO_2_ nanotube arrays with a length of up to 4.4 µm were successfully obtained in the F^−^ -containing acidic electrolyte. Meanwhile, in an alkaline solution, no nanotubes formed on the Ti foil. In addition, the viscosity and water content of the electrolyte have an important effect on the nanotubes’ morphology. For example, Zhang et al. [8] found that TiO_2_ nanotubes obtained in different viscous organic electrolytes at 20 V could exhibit different surface morphologies. “Honeycomb” shape TiO_2_ nanotubes with an inner pore diameter of 40 nm were obtained in ethylene glycol/water (99:1 vol.%), while the so called “bamboo-type” nanotubes having an average outer pore diameter of 120 nm were obtained in glycerol/water (75:25 vol.%). In addition, the effect of the water content of the electrolytes on the morphology of the nanostructures was also studied by Fraoucene et al. [15]. It was found that honeycomb and porous nanostructures were obtained on the surface of the α and β phase region in the Ti6Al4V alloy when the water content was lower than 15 wt.%, while self-organized TiO_2_ nanotubes with an inner diameter ranging from 97 nm to 206 nm were obtained when the water content was increased to 20 wt.%. In another study, TiO_2_ nanotubes were fabricated by anodization using aqueous acidic electrolytes with carboxymethylcellulose (CMC). It was found that the addition of CMC promoted the good self-organization of nanotubes, and that the inner diameters of the nanotubes strongly depended on the anodization potentials. The inner diameter was up to about 100 nm at 20 V, and the smallest diameter of about 9.5 nm was successfully obtained at a potential under 10 V [16].

Recently, many researchers have reported that nanostructured oxide films were successfully fabricated on binary or complex titanium alloys, such as TiAl [17], TiTa [18], Ti6Al4V [19,20] and Ti0.3Mo0.8Ni [21]. In this work, we investigate for the first time the synthesis of a self-ordered oxide grown on a Ti10Ni5Si alloy. Because of the different oxidation behavior of Ti, Ni and Si, it is interesting to study the behavior of the trinary TiNiSi alloy anodized in different NH_4_F-based electrolytes. We systematically investigate the effects of various electrochemical anodization parameters, including the electrolyte viscosity, water content, anodization potential and anodization time, on the growth and surface morphologies of Ti-Ni-Si-O nanostructures. In addition, we propose the formation mechanism of different nanostructures. 

## 2. Materials and Methods

**Figure 1:**
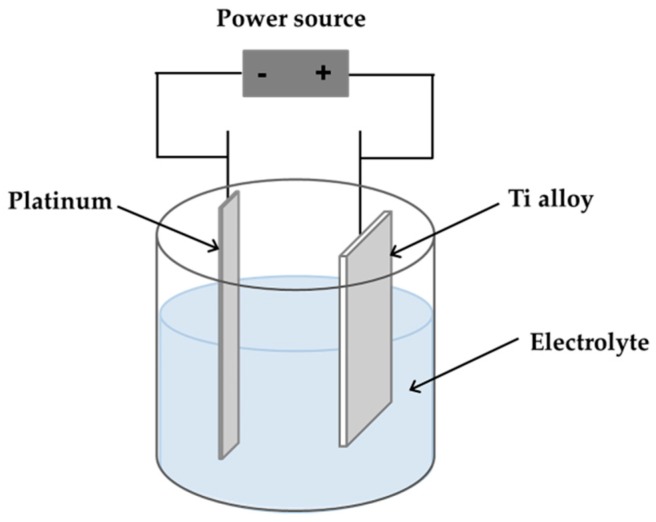
Schematic illustration of the anodization equipment.

As-cast titanium alloy (Ti10Ni5Si) plates with a dimension of 20 mm × 10 mm × 1 mm were abraded successively from 300 to 2000 grit grades with SiC papers, ultrasonically cleaned in acetone and ethanol, rinsed with deionized water, and finally dried in air. The electrochemical anodization was conducted in a conventional two-electrode system using a pulse power source (SOYIDM, Shanghai Suoyi Electronic technology Co., Ltd., Shanghai, China). The as-cast titanium alloy was used as the working electrode, and a platinum sheet was used as the cathode electrode. The schematic illustration of the anodization equipment is shown in Figure 1. To study the effect of electrolytes on the anodic oxide layer, anodizations at potentials of 30 V–50 V were carried out in the electrolytes containing ammonium fluoride (NH_4_F): a pure organic electrolyte of ethylene glycol and/or a glycerol electrolyte, as well as an organic electrolyte with a small amount of deionized water. Table 1 shows the compositions of different electrolytes, denoted as EL-1, EL-2, EL-3, and EL-4, respectively. The anodization time ranged from 60 min to 120 min. All of the anodization experiments were performed at 20 °C.

The surface and compositions of the Ti10Ni5Si alloy as well as of the anodized samples were investigated using scanning electron microscope (SEM, FEI SIRION 200, Hillsboro, OR, USA) and energy dispersive spectroscopy (EDS, INCA X-ACT, Oxford, UK). The phase structures of the alloy were examined by X-ray diffraction (XRD, Rigaku Ultima IV, Tokyo, Japan) measurements using a diffractometer with Cu Kα radiation (λ = 1.54206Å) over a scan range (2θ) of 10–80°.

## 3. Results and Discussion

**Figure 2:**
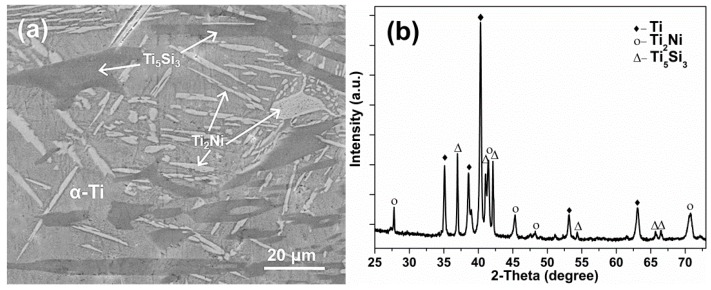
(**a**) SEM image and (**b**) XRD pattern of the Ti10Ni5Si alloy.

Figure 2a presents the typical SEM images of the Ti10Ni5Si alloy. Clearly, three different phase structures were found in the alloy: α-Ti in the gray region, the Ti_2_Ni phase of the bright strip-like and block-like region, and the Ti_5_Si_3_ phase in the dark region. The corresponding chemical compositions are summarized in Table 2. The phase structures of the alloy were also confirmed by an XRD analysis. As shown in Figure 2b, the diffraction peaks at 41.5°, 45.4° and 70.8° corresponded to the (511), (440) and (822) crystal planes, respectively, which could be assigned to the Ti_2_Ni phase [22,23]. Meanwhile, the peaks at 36.7°, 40.8° and 42.5° corresponded to the (210), (211) and (112) crystal planes of the Ti_5_Si_3_ phase [24,25]. The different phases exhibited different anodization behaviors, which will be discussed in the next section.

**Figure 3:**
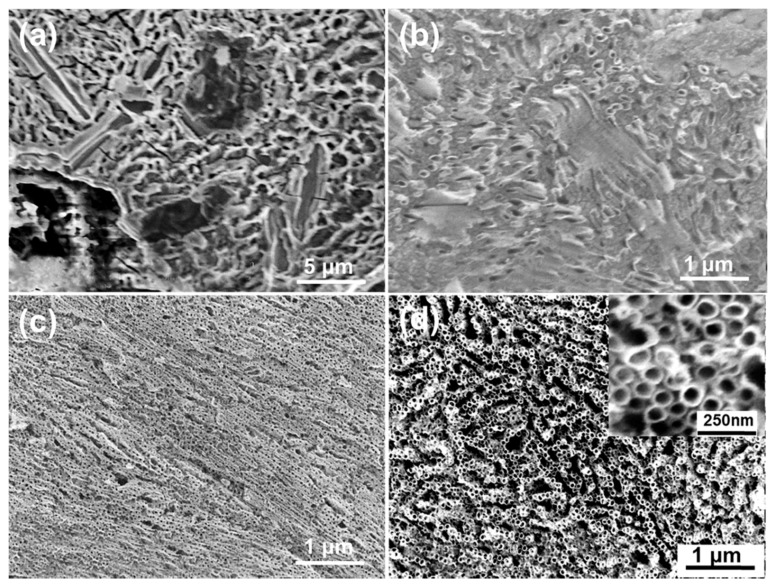
Top-view SEM images of the anodic oxide layer obtained from different electrolytes. (**a**) EL-1, (**b**) EL-2, (**c**) EL-3 and (**d**) EL-4. The anodization was performed at 30 V for 90 min.

In order to study the effect of the electrolytes on the surface features, the anodization was performed at 30 V for 90 min in different NH_4_F-containing electrolytes. Figure 3 shows the top-view SEM images of the anodic oxide layer grown in the α-Ti region of the alloy. Obviously, the surface morphology obtained in the various electrolytes exhibited dramatic differences. As shown in Figure 3a, when using the pure ethylene glycol electrolyte, only etching pits and cracks were observed on the oxide layer, instead of a nanotube or nanopore structure. These cracks might be caused by the surface stress, which originated during the oxidization process [26]. When using the pure glycerol electrolyte, a random generation of disordered small pores was found on the oxide layer (Figure 3b). The water content in the electrolyte was beneficial to the pores’ expansion. As shown in Figure 3c, in the glycerol electrolyte that had a small amount of water, the disordered small pores expanded to self-ordered nanotubes with considerably small inner diameters. Figure 3d shows the self-organized nanotubes grown in the ethylene glycol/glycerol solutions with a small amount of water. Remarkably, the average inner diameter of the nanotubes increased to 55 nm and the wall thickness to about 12 nm.

Generally, the formation mechanism of TiO_2_ nanotubes is considered to be a competition between the growth of the anodic TiO_2_ layer and the chemical dissolution of TiO_2_ by an F^−^ reaction [27,28]. Correspondingly, the overall reactions for this growth process can be described as [29]:

Ti + 2H_2_O → TiO_2_ + 4H^+^ + 4e (1)

TiO_2_ + 4H^+^ + 6F^−^ → [TiF_6_]^2−^ + 2H_2_O (2)

At first, the initial reaction rate of the electrochemical oxidation is much faster than that of the chemical dissolution, leading to the formation of an oxide layer growth on the metal surface. However, the oxide layer is constantly attacked by the F^−^ ions in the electrolyte to form [TiF_6_]^2−^ species. These [TiF_6_]^2−^ species strengthen the local electric field at the bottom, resulting in a field-enhanced chemical dissolution of the oxide layer and thus promoting the formation of nanotubes. Additionally, the local acidification also increases the chemical dissolution rate. Finally, a delicate balance between the electrochemical oxidation rate of the oxide top and the chemical dissolution rate of the oxide bottom is reached, and this is when a stabilized nanostructure is obtained. 

Obviously, the presence of fluoride ions and the ion diffusion rate play an important role in the surface morphology of the anodized oxide layer. The decisive factor for the electrolyte diffusion control, i.e., the diffusion constant, depends on the dynamic viscosity of the electrolytes, which can be described by the Stokes-Einstein equation [8]:

D = (KT)/(3πηd)(3)

where K, T, η and d respectively represent Boltzmann’s constant, the absolute temperature, the dynamic viscosity and the diameter of a spherical body.

The dynamic viscosity of glycerol (1.5 Pas) is about 71 times that of ethylene glycol (0.021 Pas) [8]. A high viscosity decreases the migration rate of ions in the electrolyte, and consequently more ions have enough time to take part in the interactions. Furthermore, the electrolyte dynamic viscosity is in an inverse proportional relationship to the electrolyte conductivity, according to Walden’s rule [30]: the higher the dynamic viscosity, the lower the electrolyte conductivity. Therefore, in the ethylene glycol electrolyte, the chemical etching rate of the oxide layer was much higher than the growth rate due to the low dynamic viscosity and high electrolyte conductivity, leading to the serious etching pits on the surface. On the other hand, in the glycerol electrolyte, the process of chemical etching was too slow to form ordered nanotubes. One can conclude that the EL-4 electrolyte was the most suitable electrolyte for forming ordered self-organized nanotubes.

**Figure 4:**
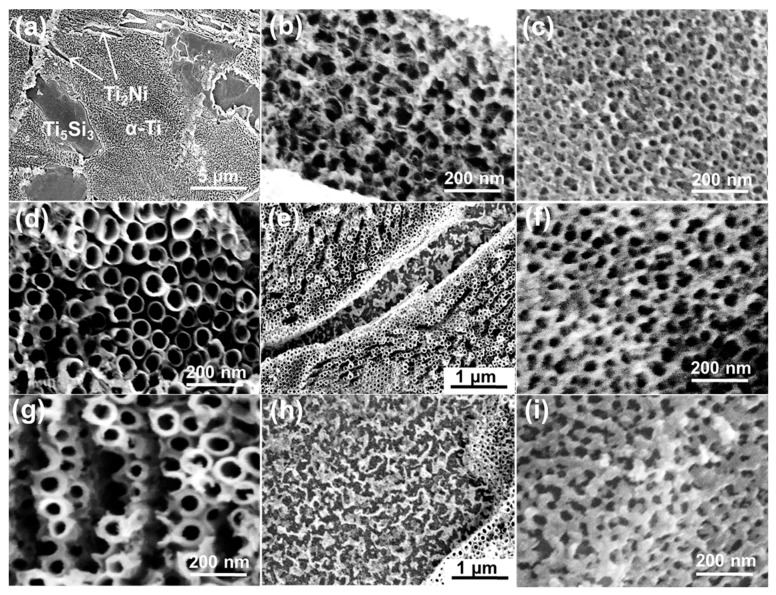
Top-view SEM images of the nanostructures grown in the α-Ti phase, Ti_2_Ni phase and Ti_5_Si_3_ phase regions at different potentials. (**a**–**c**) 30 V, (**d**–**f**) 40 V, and (**g**–**i**) 50 V. All anodization experiments were carried out in EL-4 electrolyte for 90 min.

A phase-dependent anodization has been observed in many titanium alloys, such as Ti6Al4V [19], Ti6Al7Nb [31] and Ti5Ni [32]. For the ternary Ti10Ni5Si alloy, different phases perform different behaviors during the anodization. Figure 4 shows the top-view SEM images of the Ti-Ni-Si-O nanostructures grown in the α-Ti phase region, Ti_2_Ni phase region and Ti_5_Si_3_ phase region at different potentials for 90 min. All the anodization experiments were carried out in the EL-4 electrolyte. Figure 4a shows the low-magnification SEM image of nanostructures which grew in the α-Ti phase, Ti_2_Ni phase and Ti_5_Si_3_ phase regions (shown in Figure 2a). The self-organized nanotubes grown in the α-Ti phase region at 30 V were discussed in detail (see Figure 3d). From Figure 4b,c, one can see that the Ti_2_Ni phase region and Ti_5_Si_3_ phase region could only lead to Ti-Ni-Si-O nanopores, with an average inner pore diameter of 50 nm and 20 nm, respectively. A previous report [33] found that during the anodization of Ni, Ni brought a higher polarization current (about five times higher than that of the Ti sample), indicating a faster and more severe chemical dissolution than that of Ti. Similarly, Si could be dissolved faster than Ti due to its high electronegativity (1.8). Therefore, nanopores rather than nanotubes formed in the Ti_2_Ni phase and Ti_5_Si_3_ phase regions due to the rapid chemical dissolution rate.

With the increase of the anodization potential to 40 V, the F^-^ ion mobility was further accelerated, leading to a faster chemical dissolution rate of the TiO_2_ oxide layer, which in turn resulted in a larger nanotube diameter. Markedly denser and more vertically oriented nanotube arrays were found in the α-Ti phase region (Figure 4d). These nanotube arrays were highly aligned compared to those formed at 30 V. The average inner diameter and wall thickness reached 70 nm and 12 nm, respectively. As shown in Figure 4e, only etching pits remained in the Ti_2_Ni phase region. From Figure 4f, we see that the average inner pore diameter of the nanopores grown in the Ti_5_Si_3_ phase region (about 25 nm) was slightly larger than that of the nanopores obtained at 30 V (see Figure 4c). At 50 V, remarkable damage was found in the nanotubes (Figure 4g). As expected, etching pits also remained in the Ti_2_Ni phase region (Figure 4h). The nanopores collapsed in the Ti_5_Si_3_ phase region, with an increased wall thickness. This may be explained by the theory that the higher anodization potential accelerated the oxidation and field-assisted dissolution rate, leading to a greater wall thickness. One can conclude that the anodization potential affected not only the surface features of the anodic oxide layer, such as the nanotube diameter, nanotube density and nanopore diameter, but also affected the nanotube arrangement. The best nanotube arrangement was obtained at 40 V.

**Figure 5:**
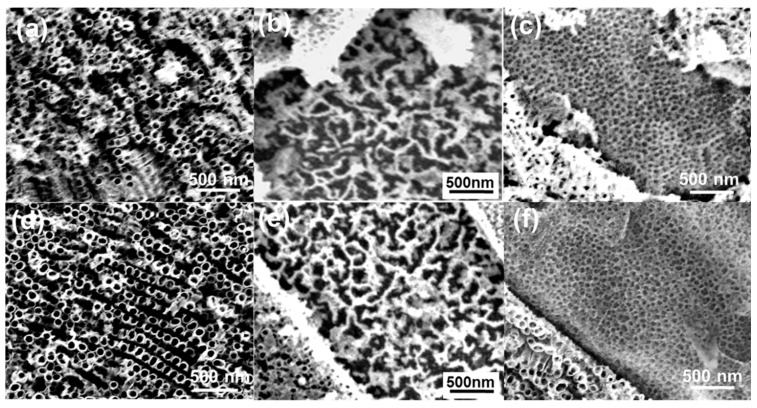
Top-view SEM images of the Ti-Ni-Si-O nanostructures grown in the α-Ti phase, Ti_2_Ni phase and Ti_5_Si_3_ phase at 40 V for different times. (**a**–**c**) 60 min, and (**b**–**f**) 120 min. All anodization experiments were carried out in EL-4 electrolyte.

Figure 5 shows the top-view SEM images of the nanostructures grown in the α-Ti phase, Ti_2_Ni phase and Ti_5_Si_3_ phase regions. The anodization was conducted at 40 V for different times One can see that etching pits and nanopores still formed in the Ti_2_Ni phase and Ti_5_Si_3_ phase regions, respectively (Figure 5b,c,e,f), which means that, compared to the factor of anodization time, the applied potential was the major factor affecting the surface morphologies. Compared to the nanotube obtained after 90 min of anodization (Figure 4d), the nanotubes grown at 60 min had a smaller average inner diameter of 65 nm and a larger wall thickness of about 18 nm (Figure 5a). When the anodization time increased to 120 min, the diameter and the wall thickness of the nanotubes were found to cease increasing (Figure 5d), which means that the oxide formation rate was already equal to the chemical dissolution rate. Nevertheless, obvious damage was observed on the top of these nanotubes. This might be attributed to the fact that, at the end of the anodization process, the initial oxide layer was completely dissolved, leading to the top of the nanotubes being exposed in the F^−^-containing electrolyte. The chemical dissolution consistently occurred with a slow speed at the top of the nanotubes. Undoubtedly, the top of the nanotubes gradually eroded when the anodization time was too long.

As mentioned above, highly ordered nanotube arrays could be fabricated in the most suitable anodization condition when the anodization was carried out at 40 V for 90 min in the EL-4 electrolyte. EDS analyses revealed that the nanostructures were composed of Ti, Ni, Si, and O elements. The Ni element was still rich in the Ti_2_Ni phase region, and Si was still rich in the Ti_5_Si_3_ phase region.

## 4. Conclusions

In summary, Ti-Ni-Si-O nanostructures were successfully synthesized on a Ti10Ni5Si alloy by an electrochemical anodization in electrolyte solutions containing ammonium fluoride (NH_4_F). The effects of the electrolyte viscosity, water content, anodization potential and anodization time on the formation of Ti-Ni-Si-O nanostructures were systematically investigated. When using an anodization potential of 40 V for 90 min in an ethylene glycol/glycerol electrolyte with 3 vol.% deionized water, highly aligned self-organized nanotube arrays with an average inner diameter of 70 nm and a wall thickness of about 12 nm were obtained in the α-Ti phase region of the alloy substrate. Meanwhile nanopore structures with an average pore diameter of 25 nm formed in the Ti_5_Si_3_ phase region. Only etching pits remained in the Ti_2_Ni phase region. 

## Figures and Tables

**Table 1:** Compositions of different electrolytes used in this work.

Composition	EL-1	EL-2	EL-3	EL-4
NH_4_F	0.3 M	0.3 M	0.3 M	0.3 M
Ethylene glycol	100 vol.%	-	-	7 vol.%
Glycerol	-	100 vol.%	95 vol.%	90 vol.%
Water	-	-	5 vol.%	3 vol.%

**Table 2:** Chemical compositions of the alloy determined by EDS.

Phase Region	Ti (wt.%/at.%)	Ni (wt.%/at.%)	Si (wt.%/at.%)
α-Ti	89.2/90.4	10.1/8.3	0.7/1.3
Ti_2_Ni	69.9/73.1	28.7/24.5	1.4/2.4
Ti_5_Si_3_	73.1/64.5	6.4/4.6	20.5/30.9
